# Perception of Nurses on Needs of Family Members of Patient Admitted to Critical Care Units of Teaching Hospital, Chitwan Nepal: A Cross-Sectional Institutional Based Study

**DOI:** 10.1155/2018/1369164

**Published:** 2018-06-26

**Authors:** Ishwori Khatri Chhetri, Bedantakala Thulung

**Affiliations:** Chitwan Medical College, College of Nursing, Bharatpur, Chitwan, Nepal

## Abstract

**Background:**

Critical care units' nurses should seek to develop collaborative relationships with patients' family members based on their needs and help them to cope with their distress. The objective of this study was to find out the perception of nurses on needs of family members of patients admitted to critical care units.

**Methods:**

A descriptive cross-sectional study was conducted in Chitwan Medical College Teaching Hospital among all 65 nurses working in critical care units. Ethical clearance was obtained from Chitwan Medical College Institutional Review Committee. Data were collected from March 27 to April 25, 2016, using Critical Care Family Needs Inventory (CCFNI). Obtained data were analyzed using descriptive and inferential statistics.

**Results:**

This study found that mean age of the nurses was 23.98 ± 4.05 years. More than half of the nurses had completed PCL in nursing (52.3%) and had 1-5 years of experience in critical care units (58.5%). Nurses ranked the needs for assurance as most important needs with mean percent (86.25%) followed by needs for information (78.58%), need for comfort (69.59%), needs for closeness (69%), and needs for support (64.13%). Out of 45 family needs, 81.5% of nurses perceived that knowing about patient treatment is very important for family members. Married nurses perceived the needs for support to be more important than unmarried nurses (p=0.04) whereas unmarried nurses perceived the needs for information to be more important than married (p=<0.01). There was significant difference on perception of nurses on needs of assurance with ethnicity (p=0.009) and critical care experience (p=0.04).

## 1. Introduction

The family is one of the basic units of society and has a great influence on its members. When a family member becomes ill, the illness affects the well-being of other family members, causing changes in the life of the whole family. Critical illness often occurs without warning and there is little time for patients and their families to prepare. If family members' immediate needs can be met, desirable consequences for both family members and patients can be achieved. In order to meet family member's needs, critical care units' nurses must be able to identify their needs accurately [1, 2]. Every year in the United States, approximately 20% of all deaths occur in an intensive care unit (ICU), and family members suffer from being withdrawn or withheld. Many of patients are unable to communicate because of sedation, mechanical ventilation, confusion, and comatose. This results in much of the burden of decision-making and treatment choices on the patients' family members. This may affect family members by increasing their stress levels and increasing their risk for psychological and physical symptoms [3].

In ICU an array of equipment, IV lines, medications, and sounds becomes unfamiliar to the public and this added fragile emotional stress to families and friends of admitted patient. This rush of unforeseen stimuli often leads to feelings of fear and powerlessness to family members [4]. Relatives go through traumatic experiences when a family member is admitted to critical care units which are unplanned and occur as emergencies. Relatives are not psychologically prepared for their patient's critical illness and their life becomes disorganized and disrupted adding to the stress of family members [5, 6].

The mortality rate of patients in the ICU is between 12 and 17%. In such condition, the family of the patients needs to provide physical and emotional support. It is the duty of nurses to provide clear and appropriate information and compassionate care to family members. Families constantly adapt themselves to the stressful situation so they need the emotional, informational, and instrumental support [7]. Meeting the needs of their patients' family members is an essential part and responsibilities of critical care units' physicians and nurses, who are committed to easing the pain and suffering of patients' relatives or close friends. A major task of nurses and physicians is to provide family members with the appropriate, clear, and compassionate information as they need to participate in making decisions about patients who are unable to speak for themselves [8].

Nursing care must address the needs not only of the patient, but those of the whole family. The needs of patients in intensive care and those of their families are especially complicated by the physical and emotional demands. Families experience severe stress and anxiety and may feel helpless and unable to cope. Accurate assessment of their needs is one of the first steps in providing appropriate care to ICU patients and their families [9]. The plight of family members has generated much interest in family care. A number of studies have been conducted to identify family needs in the critical care unit. Using the Critical Care Family Needs Inventory (CCFNI), developed by Molten in 1979 and revised by Leske in 1991, most studies have confirmed the following basic needs of family members as needs for information, assurance, support, closeness or proximity, and comfort [10].

Meeting needs of relatives of critically ill patients can have a positive impact on a patient's ability to cope during their hospital stay. When families have a stress, they may be unable to support the patient and transfer their stress to the patient [11]. Access to information about patient's conditions and quality relationships with nurses are high priority needs for families. Meeting these needs of the family members is a primary responsibility of physicians and nurses of critical care units. Families have expectations to fulfill the needs from healthcare providers which are commonly overlooked or become secondary to caring for the patient [12, 13]. Molter (1979) conducted an exploratory descriptive study to ascertain the needs of family members of patients who were critically ill and identified the 45 needs of family members [14].

### 1.1. Purpose of Study

The purpose of the study was to find out the perception of nurses on the needs of family members in critical care unit and to identify the difference between perceptions of nurses on needs of family members with sociodemographic variables.

## 2. Methods

### 2.1. Study Design and Period

Institutional based descriptive cross-sectional study was conducted from March 27 to April 25, 2016.

### 2.2. Study Area

The study was conducted in critical care units of Chitwan Medical College Teaching Hospital (CMCTH), Bharatpur, Chitwan, which is one of the largest district general hospitals of Chitwan with a total catchment population of about 20,000 in-patients with 3,500 patients in critical care units in 2015 (CMCTH, 2015). This hospital has ICU, Neuro ICU, and CCU and HDU as critical care units with 73 beds and deals with all types of diseases. The population selected in this study was all the nurses working in critical care units. There were a total of 65 nurses working in critical care units.

### 2.3. Participants

Participants were all the working nurses who have working experience of more than six months in critical care units in CMCTH.

### 2.4. Instrument

The self-administered questionnaire was used for sociodemographic characteristics and to assess the needs of family members, standard tool was adopted from Critical Care Family Needs Inventory (CCFNI) which was developed by Molter (1979) and revised by Leske (1991). CCFNI is composed of 45 needs statements that were rated on a Likert scale of 1 to 4 according to their importance: 1= not important, 2= slightly important, 3= important, and 4= very important. The total score was calculated by adding together the score for each of the 45 items. The minimum score was 45 and maximum score was 180. The 45 needs statements of CCFNI had been categorized into five subscales as needs for support (14 needs), needs for information (9 needs), needs for closeness and proximity (9 needs), needs for assurance (7 needs), and needs for comfort (6 needs).

Validity and reliability of the CCFNI tool have been documented. Cronbach's alpha coefficient of English Version CCFNI was 0.90 (Leske, 1991) which showed a high degree of internal consistency. The instrument has been validated in different culture and language. However, content validity of the tool in Nepalese context was established by consulting with experts of concerned area and research advisor and by the review of the related literatures. Reliability of Nepali Version CCFNI was calculated by using the data of pretest of six similar participants in critical care unit of College of Medical Science Teaching Hospital, Chitwan. Cronbach's alpha coefficient was computed which was 0.80 among 45 needs items which showed a high degree of internal consistency.

### 2.5. Data Collection Methods

A semi-structured and pretested self-administered questionnaire was used to gain data from the study participants.

### 2.6. Data Quality Assurance

Data quality was assured by using different approaches. At first adequate orientation was provided for data collection. After that 10% of the questionnaire was pretested on nurses having similar characteristics with study population in the similar area of College of Medical Sciences and Teaching Hospital (CMSTH). After pretest some questions were modified.

### 2.7. Data Processing and Analysis

Data were thoroughly checked for accuracy, completeness, and consistency. The collected data were organized and coded and entered in EPI data 3.1 and exported into the Statistical Package for Social Science (SPSS) version 20 for analysis. The results were presented in the form of frequencies and percentages by using tables.

### 2.8. Ethical Consideration

Ethical clearance was obtained from Institutional Review Committee (CMC-IRC). All the process started after getting approval letter from College of Nursing, Chitwan Medical College, and ethical clearance from Institutional Review Board. Further, the researcher submitted approved proposal along with written request letter from College of Nursing, Chitwan Medical College, to Chitwan Medical College Teaching Hospital, Chitwan, for the data collection. Permission for data collection was granted from the Hospital Director of CMCTH.

## 3. Findings of the Study

Table 1 illustrates that among total of 65 nurses, majority (92.3%) of the participants belonged to age group of 20–29 years and almost all (98.5%) were female. Majority (70.8%) of participants were unmarried. Similarly 76.9% belonged to Brahman/Chhetri Dalit. Most of the participants (95.4%) were Hindu followed by 4.6% Buddhist. More than half (52.3%) were PCL nursing and 23.1% were BN. More than half (52.3%) were working in ICU. Concerning participants' experience, majority (72.3%) of the participants had 1–5 years of total professional experience and similarly more than half (58.5%) had 1–5 years of experience of working in critical care units. Most of the participants (92.3%) did not get critical care training and only 7.7% got training. Among them who had taken critical care training, 60% had taken training 1 year back.

Table 2 shows that, among 65 respondents, most of the participants (80%) did not have past history of relatives admitted to critical care unit and 20 had relatives admitted to critical care unit. Among them who had past history of relatives admitted to critical care unit, majority (84.6%) had family members admitted before 1-5 years likewise, and 38.46% had father admitted to critical care unit in past days.

Table 3 depicts that, out of 45 individual needs, most important top five needs as perceived by nurses were as follows: “the needs of what medical treatment patient is receiving was highest priority with mean and standard deviation of 3.82 ±0.39 followed by explanation of environment, reason for various procedure, knowing details of patient's progress, and receiving detailed information about the patient with mean and standard deviation of each”.

Table 4 shows that the five least important needs as perceived by nurses were as follows: “to visit patient at any time was least important needs as followed by to be alone at any time, to have another person with relatives in ICU, availability of clergymen in hospital, and to talk the negatives feelings with mean and standard deviation of each”.

Table 5 shows that mean percentage were calculated on the five subscales of the CCFNI. The subscales of assurance ranked the highest (86.25%) and the subscale for support ranked the lowest (64.13%) needs of the family members as perceived by nurses.

Table 6 depicts that there was significant difference on perception on needs for support with marital status of respondents (*p*=0.049) in which married nurses perceived needs for support to be more important than unmarried nurses. Similarly there was statistically significant difference on perception on needs for information with marital status (*p* < 0.05) in which unmarried nurses perceived needs for information to be more important than married nurses.

Table 7 shows that there was not statistically significant difference between perception on needs of family members with professional qualification, clinical area of working, and total professional experience (*p*>0.05). There was significant difference on perception on needs of family members with critical care experience of the nurses (p=0.050) in which nurses having experience of one year and more perceived the needs for closeness to be more important than nurses having experience less than one year of experience.

Table 8 reveals relationship between the subscales of needs of family members. Among the subscales, the highest correlation was found between the needs for closeness and assurance (*r*=0.48) followed by needs for information and assurance (*r*=0.35), needs for assurance and comfort (*r*=0.33), and needs for information and closeness (*r*=0.31) and lowest between the needs for support and information (r=0.13). On average there was statistically significant difference in correlation between subscales (*p*<0.01).

## 4. Discussion

Regarding the most important needs as perceived by participants, out of the 45 individual needs of family members, 81.5% of nurses perceived the need of “knowing about the patients treatment” as most important needs of family members of critical care unit admitted patient with mean $\overset{-}{X}$=3.82±0.39. This finding is supported by the findings of the study conducted by Gundo in Malawi among 62 nurses showing that the most important need as perceived by nurses was as follows: “to know what medical treatment the patient is receiving was most important” with mean $\overset{-}{X}$=3.82±0.51 [15].

Among the subscales of needs of family members, nurses ranked the needs for assurance as most important needs with mean percent 86.25% and the subscale of needs for support with mean percent of 64.13% as the least priority needs of family members of critical care unit admitted patients. These results are not supported by available literature that report that the nurses ranked the most important needs and least important needs of family members differently among the subscales of needs of family members [6, 15, 16].

There was no difference on perception of needs of family members with age of the nurses, professional qualification, experience, and critical care training (*p*>0.05). In line with this study, a study of Gundo [15] shows that there was no significant relationship between perception of nurses on family needs with the nurses' age, year of experience, and critical care training (*p*>0.05). The married nurses perceived needs for support to be more important than unmarried nurses* (p*=0.049). Similarly unmarried nurses perceived needs for information to be more important than married nurse (*p*=009). In contrast with this study, the study conducted by Iranmanesh et al. [2, 15] shows that the perception on needs of family member is not statistically significant with the marital status of nurses (*P*>0.05).

In this study, the perception on needs for closeness is statistically significant with the nurses' experience in critical care unit (*p*=0.050) in which nurses with experience one year and more perceived the needs for closeness to be more important than nurses having experience of less than one year. This finding is similar to the study by Maxwell et al. (2007) which revealed that the subscales for needs for assurance and needs for closeness were rated highly by the nurses who have more experience in critical care unit than those who have less than one year of experience (*P*<0.05) [17].

## 5. Conclusion

On the basis of the study findings, it is concluded that nurses rank the subscale of needs for assurance as most important needs of family members and the subscales of needs for support as least important needs of family members. Nurses having more experience in critical care unit perceived the needs of family member to be more important. There is positive correlation between the subscales of needs of family members. Therefore, these needs are to be addressed by nurses to support the affected family members while providing care to the patient.

## Figures and Tables

**Table 1:** Sociodemographic characteristics of participants.

**Variables**	**Frequency**	**Percentage**
**Age ( in years) **		
20 – 29	60	92.3
≥30	5	7.7
**Sex **		
Male	1	1.5
Female	64	98.5
**Marital status **		
Married	19	29.2
Unmarried	46	70.8
**Ethnic group **		
Dalit	4	6.2
Janajati	11	16.9
Brahman/Chhetri	50	76.9
**Religion**		
Hindu	62	95.4
Buddhist	3	4.6
**Professional qualification **		
Pcl nursing	34	52.3
BN	15	23.1
B.Sc nursing	16	24.6
**Clinical area of working**		
ICU	34	52.3
CCU	11	16.9
HDU	8	12.3
Neuro ICU	12	18.5
**Total professional experience (in years)**		
<1	14	21.6
1-5	47	72.3
≥ 6	4	6.1
**Total experience of working in critical care unit (in years)**		
< 1	25	38.5
1-5	38	58.5
≥ 6	2	3.0
**Critical care training**		
Yes	5	7.7
No	60	92.3
**If yes, time of training (n=5)**		
<1 year	3	60.0
≥1 year	2	40.0

**Table 2:** Past history of nurses' relatives admitted to critical care units.

**Variables**	**Frequency**	**Percentage**
**Past history of relatives admitted to critical care units**		
Yes	13	20.0
No	52	80.0
**If yes, when admitted (n=13)**		
1 – 5 years back	11	84.6
≥ 6 years back	2	15.4
**Relation with patients (n=13)**		
Father	5	38.46
Mother	2	15.39
Grandfather	1	7.69
Grandmother	3	23.07
Elder mother	2	15.39

**Table 3:** Participants' perception of five most important needs of family members.

Needs of Family Members	Responses	
	Rank	Mean± SD
Knowing about patient treatment	1	3.82 ±0.39
Explanation of the environment	2	3.80 ±0.44
Reason for various procedure	3	3.74 ±0.47
Knowing details of patient's progress	4	3.72 ± 0.51
Receive daily information about the patient	5	3.71 ±0.45

**Table 4:** Respondents' perception of five least important needs of family members.

**Needs of Family Members**	**Responses**	
	**Rank**	**Mean± SD**
Talk about the negative feelings	41	1.82 ±0.84
Availability of clergymen in hospital	42	1.63±0.82
Have another person with relatives in ICU	43	1.60 ±0.88
Be alone at any time	44	1.48±0.75
Visit patient at any time	45	1.34±0.69

**Table 5:** Participants' perception score on five subscales of needs of family members.

**Subscales of Needs of Family Members**	**Responses **	
	**Rank**	**Percentage**
Needs for Assurance	1	86.25%
Needs for Information	2	78.58%
Needs for Comfort	3	69.59%
Needs for Closeness	4	69.00%
Needs for Support	5	64.13%

**Total**		

**Table 6:** Differences on perception of nurses on needs of family members according to sociodemographic variables.

**Variables**	**No.**	**Perception on Needs of Family Members**				
		**Mean**±**SD**				
		**Needs for Support**	**Needs for Information**	**Needs for Closeness**	**Needs for Assurance**	**Needs for Comfort**
**Age ( in years)**						
20 – 29	60	34.56±5.01	28.50±2.86	24.73 ±3.04	24.05 ±3.07	16.65±2.92
≥30	5	35.40±2.88	25.80±4.14	26.40 ±1.64	25.40±2.70	17.40±.89
***p*-value** **∗**		0.716	0.054	0.294	0.345	0.572
**Marital status**						
Married	19	36.47±5.15	26.78±3.20	24.26±2.51	24.26±3.29	16.31±2.51
Unmarried	46	33.86±4.59	28.91±2.75	25.08±3.14	24.10±2.97	16.86±2.94
***p*–value** **∗**		**0.049**	**0.009**	0.315	0.854	0.476
**Ethnicity**						
Dalit	4	30.75±4.50	29.25±2.06	25.25±.957	24.75±1.89	14.00±2.44
Janajati	11	32.63±4.80	26.63±3.55	23.63±3.58	21.63±3.04	16.09±1.97
Brahman/Chhetri	50	35.38±4.72	28.58±2.89	25.08±2.91	24.66±2.88	17.06±2.90
***p* –value** **∗** **∗**		0.060	0.126	0.339	**0.009**	0.081
**Religion**						
Hindu	62	34.67±4.89	28.35±3.08	24.91±2.94	24.20±3.00	16.67±2.85
Buddhist	3	33.66±5.13	27.00±0.00	23.33±4.04	23.00±4.35	17.33±2.51
***p* –value**		0.729	0.454	0.372	0.507	0.697

*S.D.: standard deviation; significance level at < 0.05. ∗Independent t-test; ∗∗ one-way ANOVA test.*

**Table 7:** Differences on perception of nurses on needs of family members according to professional characteristics.

**Variables**	**No.**	**Perception on Needs of Family Members**				
		**Mean**±**SD**				
		**Needs for Support**	**Needs for Information**	**Needs for Closeness**	**Needs for Assurance**	**Needs for Comfort**
**Professional qualification**						
Pcl nursing	34	33.82±5.01	28.11±3.16	24.50±3.03	23.52±3.42	16.20±2.74
BN	15	35.26±4.44	27.93±3.05	25.00±2.44	25.53±1.68	17.20±2.67
B.Sc nursing	16	35.75±4.91	29.00±2.78	25.43±3.36	24.18±2.90	17.31±3.07
***p* –value** **∗** **∗**		0.368	0.557	0.576	0.104	0.327
**Clinical area **						
ICU	34	35.44±4.38	28.29±2.85	24.38±2.96	24.08±3.06	16.32±2.62
CCU	11	35.45±4.76	28.90±2.58	27.00±2.14	25.00±1.84	18.45±2.94
HDU	8	31.12±6.46	27.87±3.22	24.25±4.02	24.37±3.02	16.87±3.44
Neuro ICU	12	33.91±4.58	28.00±3.95	24.58±2.27	23.41±3.94	16.08±2.50
***p* –value** **∗** **∗**		0.128	0.874	0.069	0.667	0.139
**Total experience (in years)**						
less than 1	14	33.76±4.57	28.96±3.02	24.76±3.17	24.04±3.33	17.00±2.54
1 and more than 1 year	51	35.17±5.02	27.87±2.99	26.25±2.73	24.22±2.89	16.52±2.99
***p* –value** **∗**		0.795	0.297	0.335	0.157	0.450
**Experience in critical** **care units (in years)**						
<1	25	33.76±4.57	27.71±2.94	25.76±3.17	24.04±3.33	17.00±2.54
≥1	40	35.05±5.11	29.96±3.02	24.10±2.69	24.02±2.83	16.34±2.92
***p*- value** **∗**		0.258	0.162	**0.050**	0.814	0.513
**Training about critical care**						
Yes	5	31.80 ±4.7	28.20±1.64	22.20±4.20	23.40±5.45	17.40±3.36
No	60	34.86±4.84	28.30±3.12	25.06±2.79	24.21±2.82	16.65±2.79
***P* –value** **∗**		0.178	0.948	0.434	0.569	0.572

*S.D.: standard deviation; significance level at < 0.055. ∗ Independent t-test; ∗∗ one-way ANOVA test*.

**Table 8:** Relationship between the subscales of needs of family members.

**Subscales of Needs of Family Members**	**Needs for Support**	**Needs for Information**	** Needs for Closeness**	**Needs for Assurance**	**Needs for Comfort**
Needs for Support	1				
Needs for Information	0.13	1			
Needs for Closeness	0.24	0.31^*∗*^	1		
Needs for Assurance	0.25^*∗*^	0.35^*∗∗*^	0.48^*∗∗*^	1	
Needs for Comfort	0.20	0.24^*∗∗*^	0.23	0.33^*∗∗*^	1

*∗∗ Correlation is significant at the 0.01 level (2-tailed). ∗ Correlation is significant at the 0.05 level (2-tailed)*.
